# A field-scale remediation of residual light non-aqueous phase liquid (LNAPL): chemical enhancers for pump and treat

**DOI:** 10.1007/s11356-021-14558-2

**Published:** 2021-06-03

**Authors:** Paolo Ciampi, Carlo Esposito, Giorgio Cassiani, Gian Piero Deidda, Paolo Rizzetto, Marco Petrangeli Papini

**Affiliations:** 1 Department of Earth Sciences, Sapienza University of Rome, Piazzale Aldo Moro 5, 00185 Rome, Italy; 2 Department of Geosciences, University of Padua, Via Gradenigo 6, 35131 Padua, Italy; 3 Department of Civil and Environmental Engineering and Architecture, University of Cagliari, via Marengo, 2, 09123 Cagliari, Italy; 4 Italian Air Force, Logistic Headquarter Viale dell’Università, 4, 00185 Rome, Italy; 5 Department of Chemistry, Sapienza University of Rome, Piazzale Aldo Moro 5, 00185 Rome, Italy

**Keywords:** Hydrocarbon contamination, Hydrogeophysical monitoring, Light non-aqueous phase liquid desorption, Pilot test, Contaminant remediation, Residual hydrocarbons

## Abstract

The remediation of petroleum-contaminated soil and groundwater is a challenging task. The petroleum hydrocarbons have a long persistence in both the vadose zone and in the aquifer and potentially represent secondary and residual sources of contamination. This is particularly evident in the presence of residual free-phase. Pump-and-treat is the most common hydrocarbon decontamination strategy. Besides, it acts primarily on the water dissolved phase and reduces concentrations of contaminants to an asymptotic trend. This study presents a case of enhanced light non-aqueous phase liquid (LNAPL) remediation monitored using noninvasive techniques. A pilot-scale field experiment was conducted through the injection of reagents into the subsoil to stimulate the desorption and the oxidation of residual hydrocarbons. Geophysical and groundwater monitoring during pilot testing controlled the effectiveness of the intervention, both in terms of product diffusion capacity and in terms of effective reduction of pollutant concentrations. In particular, non-invasive monitoring of the reagent migration and its capability to reach the target areas is a major add-on to the remediation technique. Most of the organic contaminants were decomposed, mobilized, and subsequently removed using physical recovery techniques. A considerable mass of contaminant was recovered resulting in the reduction of concentrations in the intervention areas.

## Introduction

The remediation of the areas contaminated by petroleum hydrocarbons and the selection of the best decontamination methods represent a growing global concern (Kuppusamy et al. 2020; Ossai et al. 2020; Verardo et al. 2021). The natural aging of petroleum hydrocarbons in contaminated sites and the water table fluctuations result in the chemical sequestration and physical entrapment of these hydrophobic compounds (Gatsios et al. 2018; Teramoto et al. 2020). This aspect is particularly relevant in the case of contamination by fuels, which are complex mixtures of hydrocarbons made of substances with significantly different chemical, physical, and biodegradation properties (Vozka et al. 2019). The progressive aging of the contaminants corresponds to a reduction of the more mobile and degradable fractions and the increase of compounds with a higher molecular weight (Tran et al. 2018). Such immobile, less volatile, less soluble, more viscous, and high molecular weight materials constitute the residual hydrocarbons, which are difficult to be mobilized by traditional extraction (i.e., pumping) technologies (Lari et al. 2019b; Ossai et al. 2020; Teramoto et al. 2020; Trulli et al. 2016). This aging or weathering can cause modifications that are necessary to consider when selecting a remediation technique and is therefore essential to the polluted siteʼs management (Lari et al. 2019a; Tang et al. 2012). The light non-aqueous phase liquid (LNAPL) recovery and contamination mitigation approaches may include hydraulic pumping (mainly to recover the LNAPL), soil vapor extraction (SVE), chemical oxidants (e.g., to reduce saturation and degrade contaminants), air sparging (e.g., to augment biodegradation and volatilization), thermal methods (e.g., to decrease LNAPL viscosity and increase volatilization), enhanced bioremediation, multiphase extraction (MPE), skimming of mobile LNAPL, and natural source zone depletion (i.e., NSZD) (Besha et al. 2018; Bortoni et al. 2019; Gatsios et al. 2018; Kuppusamy et al. 2020; Lari et al. 2019a; Lari et al. 2020; Ossai et al. 2020; Sharma et al. 2020; Verardo et al. 2021; Xie et al. 2020; Yao et al. 2020). Among the physical extraction-based remediation techniques, the pump-and-treat is traditionally the most commonly used approach for treating contaminated groundwater (Brusseau 2019; Teramoto et al. 2020). While the initial phase of pump-and-treat systems typically achieves a rapid reduction of light non-aqueous phase liquids (LNAPLs) aqueous concentrations, its long-term effectiveness diminishes, and the system often reaches asymptotic conditions (Truex et al. 2017). Further operations of the system provide small incremental benefits in treating soil or groundwater contamination, often not achieving the regulation cleaning goals and being operationally long and expensive (Lari et al. 2019b). Pump-and-treat efficacy tends to plateau as a result of a variety of factors such as (a) hydrocarbon distribution through zones of differential matrix permeability and (b) the presence of slowly dissolving smeared free phase and/or adsorbed hydrocarbon contamination (Lee et al. 2001; Lari et al. 2019a; Teramoto et al. 2020). Miscible solvents and surfactants can act as chemical enhancers for pump-and-treat: the petroleum hydrocarbon mass can be removed using reagent to enhance recovery of sorbed-phase or smeared hydrocarbon (McCray et al. 2011; Sharma et al. 2020). The hydrocarbons are made available in the dissolved or lower viscosity phase, to enhance the recoverability of the product in a separate phase, allowing a subsequent rapid and effective physical recovery (Birnstingl et al. 2014; Lari et al. 2019b). Characterization efforts are critical for determining the applicability, deployment, and efficacy of remediation technology. From the implementation perspective, field applications should continuously emphasize adequate site characterizations for a proper remedial design (Lari et al. 2020; Suthersan et al. 2016). Verification of amendment distribution in soils should be part of the performance monitoring (Fan et al. 2017).

A possible strategy for this monitoring relies on the use of non-invasive geophysical techniques to visualize the time-lapse distribution of reagents. Thus, the resulting physical changes due to the injected solution may be measured using physical methods. The use of repeated geophysical measurements to highlight changes in the system‘s condition is the state-of-the-art for several hydrological applications (Cassiani et al. 2006; Deiana et al. 2008; Morita et al. 2020; Perri et al. 2012; Haaken et al. 2017) but rarely implemented at contaminated sites, particularly during remediation activities (Cassiani et al. 2014; Perri et al. 2020). Although geophysical experiments and field studies provided valuable insights on the behavior of contaminants, the results are still ambiguous, leading to widely divergent explanations (Atekwana and Atekwana 2010; Deng et al. 2020; Hort et al. 2015). These methods can provide potentially critical information on where and how in situ remediation actions affect different portions of the subsurface, as an effect of subsoil hydraulic heterogeneity (Vereecken et al. 2006). The physical variable of interest, i.e., the electrical resistivity, is strongly linked to state variables of key environmental interest (Lesmes and Friedman 2005). In this regard, the joint modeling of geological-geophysical data enabled Ciampi et al. (2019a) to discretize the electrical response and to track the product diffusion resulting from reagent injection into a heterogeneous, dense non-aqueous phase liquid (DNAPL)-contaminated aquifer. The present paper, unlike the previous one, aims to unmask the decontamination dynamics induced by the injection of amendments in the context of an LNAPL-contaminated site. The geophysical-chemical cross-analysis can potentially explain the removal mechanisms of residual hydrocarbons, by identifying the different fractions of product involved in the degradative processes (Ossai et al. 2020). Effective remediation of a site contaminated with hydrocarbons requires a sound understanding of regulatory issues, technology options, and the siteʼs hydrogeology (Lari et al. 2020). In this context, the challenge to be faced is to simultaneously integrate the information relating to the hydrogeophysical sphere in all its dimensions (Harris et al. 2004; Teramoto et al. 2020; Verardo et al. 2021). A cross-disciplinary geodatabase and an interactive model become the instruments for managing and analyzing multi-source data (Ciampi et al. 2019a). The hydrogeological complexity, geophysical manifestation, and contamination or decontamination processes are caught by information sharing, knowledge convergence, and high-resolution depiction of environmental diversity (Ciampi et al. 2019b). In this direction, the present work presents a case study that dealt with the application of an innovative technology to remediate a site contaminated with petroleum hydrocarbons. This paper focuses on a field injection of the PetroCleanze™ product (Regenesis, San Clemente, CA) for enhancing and extending the effectiveness of physical extraction systems. The technology combines in situ chemical oxidation (ISCO) and enhanced desorption to treat bound hydrocarbon and LNAPL (Besha et al. 2018; Wang et al. 2013). PetroCleanze™ is a two-part reagent, which targets sorbed-mass and residual NAPL, bringing each into the soluble and recoverable phase from where they may then be extracted through pumping systems (Sharma et al. 2020). This strategy was here verified through a pilot test, to evaluate the possible scaling up of the process. The pilot test, which was properly orchestrated via a multidisciplinary and multitemporal data management model, was assessed in terms of yield during the implementation process. Electrical resistivity tomography (ERT) monitoring and groundwater sampling were performed to evaluate the effectiveness of the intervention, both in terms of product diffusion capacity and in terms of effective reduction of pollutant concentrations. The near real-time observation of decontamination dynamics at the field scale can represent an added value to interpret the spatial and temporal physio-chemical changes during the remediation process, explaining the contaminant-geophysical behavior. The case study presents possibilities for optimizing LNAPL contaminant removal since it is substantially unrecoverable using traditional remediation technologies at long-term polluted sites.

## Materials and methods

The study site is a large airport area (NATO Military Base of Decimomannu) located in Sardinia (Italy), where about ten years ago a jet fuel spill occurred due to leakage of a transfer pipeline around the fuel tanks. The detected contamination, despite being mainly caused by a single spill, is quite extensive and has been the subject of years of pump-and-treat intervention (Trulli et al. 2016). This is operational.

The Regenesis PetroCleanze^TM^ was employed to develop an in situ enhanced chemical desorption strategy. The main technological functionality of the product is to enhance the desorption of hydrocarbons adsorbed to saturated soils or at the capillary fringe, and the product's recoverability as a separate phase (Sharma et al. 2020). The application of the product is aimed at making the hydrocarbons available in the dissolved phase, allowing a subsequent rapid and effective physical recovery (Birnstingl et al. 2014).

Pilot testing occurred in two different areas, by a direct application at existing wells and reactivating the pump-and-treat system a few days later. The chosen areas are characterized by two geological scenarios that are representative of the site’s conditions. In addition, the two zones are close to the source of historical pollution and have been strongly impacted by contamination.

From the characterization phase to the application of the treatments, the processing of a vast volume of heterogeneous data accompanied the entire remediation process (Suthersan et al. 2016). An automated knowledge management and analysis dashboard containing information relating to geological, geophysical, hydrological, and chemical fields was employed to archive and coordinate multi-thematic data. The 4D multidisciplinary geodatabase (which takes into account the time factor) held the role of an effective “near real-time” decision support system (DSS), which manages and releases data from site characterization to technique application (Ciampi et al. 2019a; Huysegoms and Cappuyns 2017). The digital and thematic database constitutes a data source used for the modeling and the editing of georeferenced information (Artimo et al. 2008). The interpretation of the resulting hydrological-geophysical model and the selection of remediation solutions were subsequently accomplished using a multiscale and multiphase methodology. (Ciampi et al. 2019a). The essential hydrogeological characteristics of the site at full scale were collected and employed to populate the model in the first step. The research centered on the pilot test areas in the final stage, with higher resolution, to examine in depth the effects of geological complexity and chemical mechanisms in the intervention sector, beginning with the multidisciplinary conceptual model derived from the first step. (Ciampi et al. 2019b). The RockWorks 17 application was employed to recreate the hydrogeological 3D model (Lekula et al. 2018). This software enables the acquisition, analysis, visualization, and integration of information from geo-referenced data. The geological, geophysical, and hydrochemical variables were interpolated and modeled during the data integration and analysis procedure (Kaliraj et al. 2015; Safarbeiranvnd et al. 2018). Geologic-geophysical data and chemical analyses performed on water samples represent the variables involved in the modeling activities. The stratigraphic sequence was reconstructed based on data derived from 85 boreholes. Stratigraphic logs reach depths ranging from 10 m to 26 m and cover an investigation area of about 265000 m^2^. A piezometric network consisting of 62 monitoring points was installed on the site. Piezometers completely intercept contaminated groundwater, generally reaching a depth of 10 m from the ground level. Data concerning chemical analyzes of water sampled and the presence of supernatant product from 2012 to 2018 are available. Dynamic and interactive extraction, both in time and space, of multi-source data from the multi-modality data source and joint model aimed to support decision-making (Lekula et al. 2018). The complete multi-temporal and multidisciplinary characterization helped the selection of a remediation technology. The joint management of geological and hydrochemical data oriented the location of the interventions at the field scale. Following an accurate reconstruction of the geochemical peculiarities, a field test was designed to optimize the operating conditions.

**Fig. 1:**
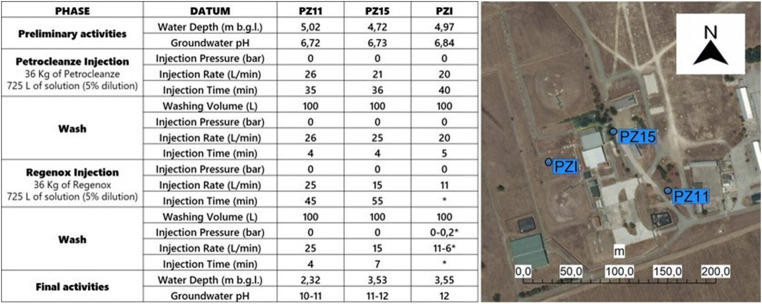
Configuration of pilot test and location of injection piezometers (PZI, PZ15, PZ11)

The pilot test aimed to assess the potential mobilization of sorbed-mass and residual LNAPL (McCray et al. 2011; Sharma et al. 2020). The remediation strategy involves the injection of reagents into the aquifer through piezometers. The reagents consist of two parts: a desorbent part (PetroCleanze™) and an oxidizing part (Regenox™, Regenesis, San Clemente, CA). The desorbed fractions can be partially oxidized but mainly physically removed by pumping. Partial oxidation intended to “break” the longest hydrocarbon chains, making the hydrophobic contaminants (slightly degradable) more soluble and easily degradable (Besha et al. 2018; Cheng et al. 2017). The injection of the parts constituting the reagent was carried out during various phases of implementation of the test at the field scale. The injections were performed at the three points of the piezometric network. Figure 1 presents the stages and the configuration of the pilot test, in terms of quantity of injected product and injection pressure or rate of the different reagents.

Groundwater sampling followed three different phases of implementation of the field test (i.e., pre-injection, post-injection, and after pumping activities). The water samples were subjected to gas chromatography and mass spectrometry (GC-MS) analysis to obtain the chemical speciation of the hydrocarbons (Fiorenza et al. 2000).

In addition to monitoring and laboratory activities, time-lapse geophysical investigations played a specific role in keeping track of cleanup mechanisms. ERT was cased in monitoring the remediation process (e.g., Chambers et al. 2010; Ciampi et al. 2019b), at injection points. The aim of the field application during the pilot test was to check the process performance and the extent of the treatment, which may differ based on the siteʼs geological features. Time-lapse changes in observed electrical resistivity of the subsoil are likely to be related to the presence of injected solutions if these have an electrical conductivity different from that of native groundwater. Similar approaches have been used in a variety of cases with different specific goals, but always ultimately linked to identifying pathways of solute migrations in the subsurface (e.g., Cassiani et al. 2006; Perri et al. 2012, 2018; Camporese et al. 2015; Busato et al. 2019).

The analytical monitoring of the piezometric network is intended to weigh the yield of the remediation technology used, thus indirectly assessing the performance and contribution of the intervention methodology. The pilot test was designed to ameliorate the layout of the intervention, to check its efficiency, and to calibrate the preliminary design of an optimized full-scale intervention.

## Results

### The 3D geological model

**Fig. 2:**
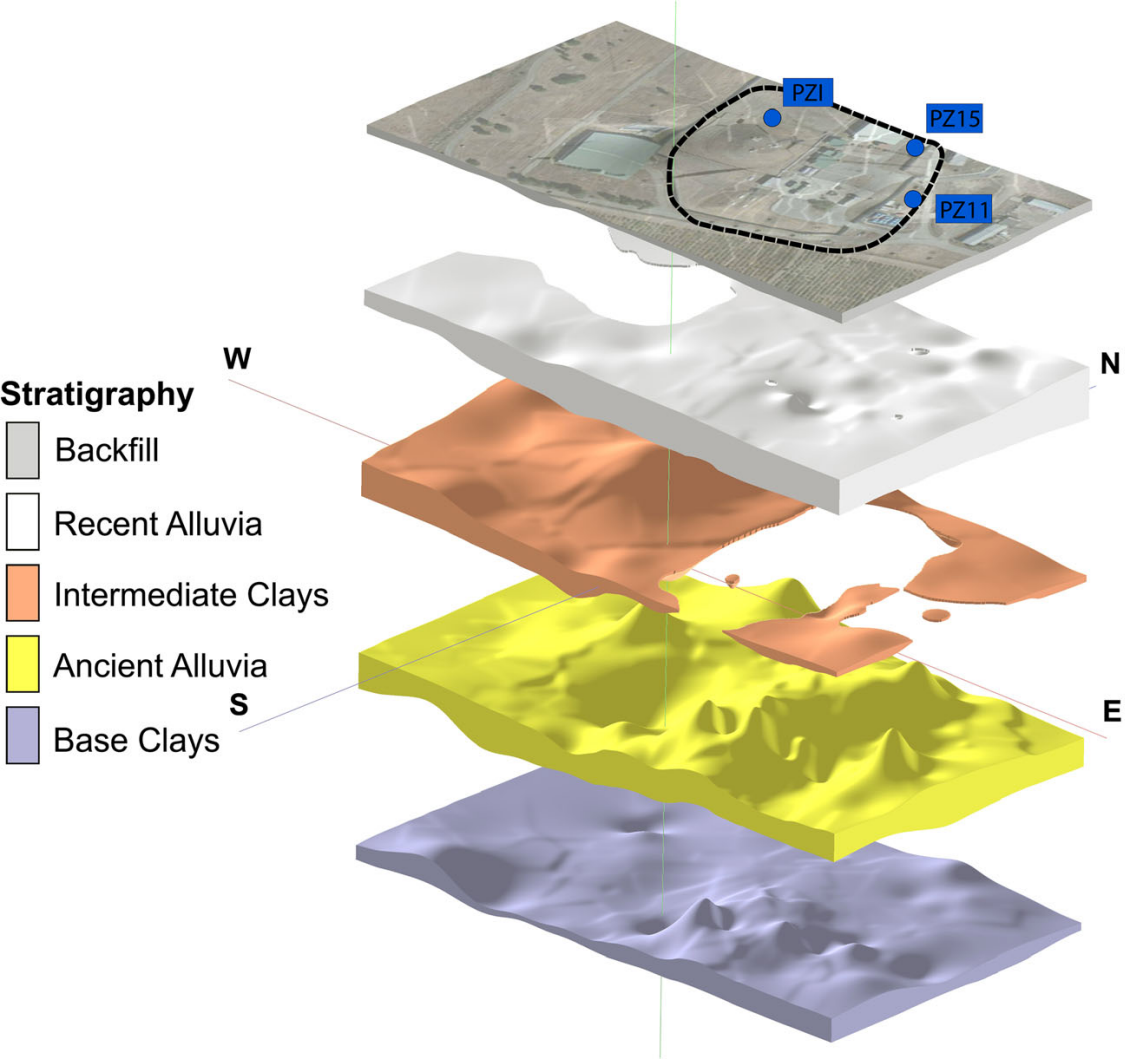
Three-dimensional geological model (with vertical exaggeration) of the Decimomannu military airbase and location of injection points (PZI, PZ11, PZ15). The dashed line identifies the area used for the storage of fuel tanks

In the spill area, the most recent deposits are related to a Plio-Quaternary depositional sequence of alluvial sediments (Bini 2013), organized in two macro-levels: an upper (and more recent) level is characterized by gravels and sands with the presence of fine fraction (recent alluvia), extending to maximum depth between 4 and 6 m, and a lower level featured by gravel and sand in a silty-clay matrix (ancient alluvia), reaching a depth between 8 and 10 m. The two levels have a highly variable thickness and are separated by a discontinuous horizon of sandy-gravelly clays with hazelnut color (intermediate clays) of ca. 1–2-m thickness. The whole sequence overlies a thick layer of clays and silty clays (base clays); both the base and the intermediate clays possess hydrogeological characteristics of an aquiclude and aquitard, respectively (Orozco et al. 2021). The latter reaches its maximum thickness in the western sector while locally disappears to the east, where the mixing groundwaters hosted in the two aquifers (recent and ancient alluvia) occur. The local subsoil is characterized by the alternation of fine- and coarse-grained materials hosting a groundwater circulation, with a mean depth to the water table of about 5 m showing significant fluctuations throughout the year. The geological model presented in Fig. 2 uses a vertical exaggeration factor and a representation offset between the different stratigraphic levels to mark the lithological steps.

The 3D stratigraphic reconstruction reveals a different geologic context for the east and west portions of the model. In the eastern sector, the intermediate clays are absent while the alluvia are preponderant. In the western sector, the intermediate clays reach their maximum thickening at the site and the recent alluvia disappear. This inevitably affects the hydraulic properties of the system, delineating a more permeable zone to the east and a less permeable zone to the west.

### Evolution of groundwater contamination

**Fig. 3:**
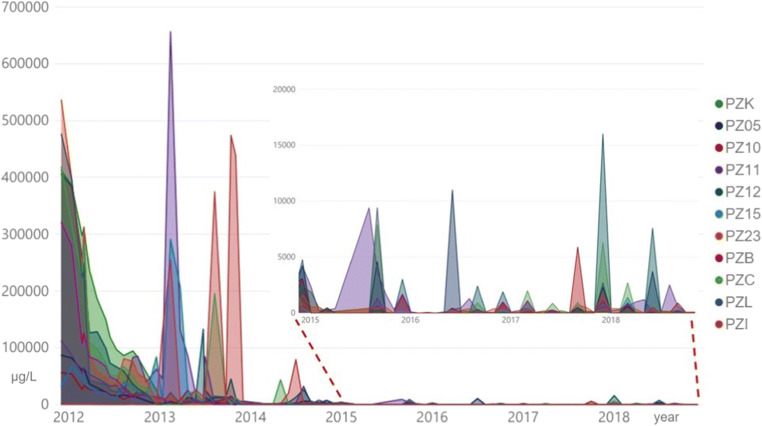
Concentration of total petroleum hydrocarbons detected in the piezometers located inside the area used for the storage of the fuel tanks over time

The reconstruction of the evolution of groundwater contamination status illustrates (1) the effects of pump-and-treat intervention over time, (2) the reduction of the total contaminant mass, and (3) a narrowing of the contaminant plume that progressively reaches an asymptotic trend (Truex et al. 2017) (Fig. 3).

The significant decrease in hydrocarbon concentrations over time suggests the aging of the contamination primary source (Atekwana and Atekwana 2010; Tran et al. 2018). This assertion is confirmed by the total absence of volatile organic compounds, such as benzene, ethylbenzene, toluene, and xylene, in the last years of monitoring (not shown here) (Trulli et al. 2016; Verardo et al. 2021). The recent measurement of hydrocarbon concentrations in groundwater reveals the presence of residual contaminants (Gatsios et al. 2018; Teramoto et al. 2020). They are considered not movable with the pump-and-treat technology being performed, due to the presence of phases adsorbed mainly to the less permeable portions of the aquifer and the occasional occurrence of LNAPL product in separate phase (limited thickness difficult to remove) (Lee et al. 2001; Lari et al. 2019a; Lari et al. 2019b).

### Pilot testing

**Fig. 4:**
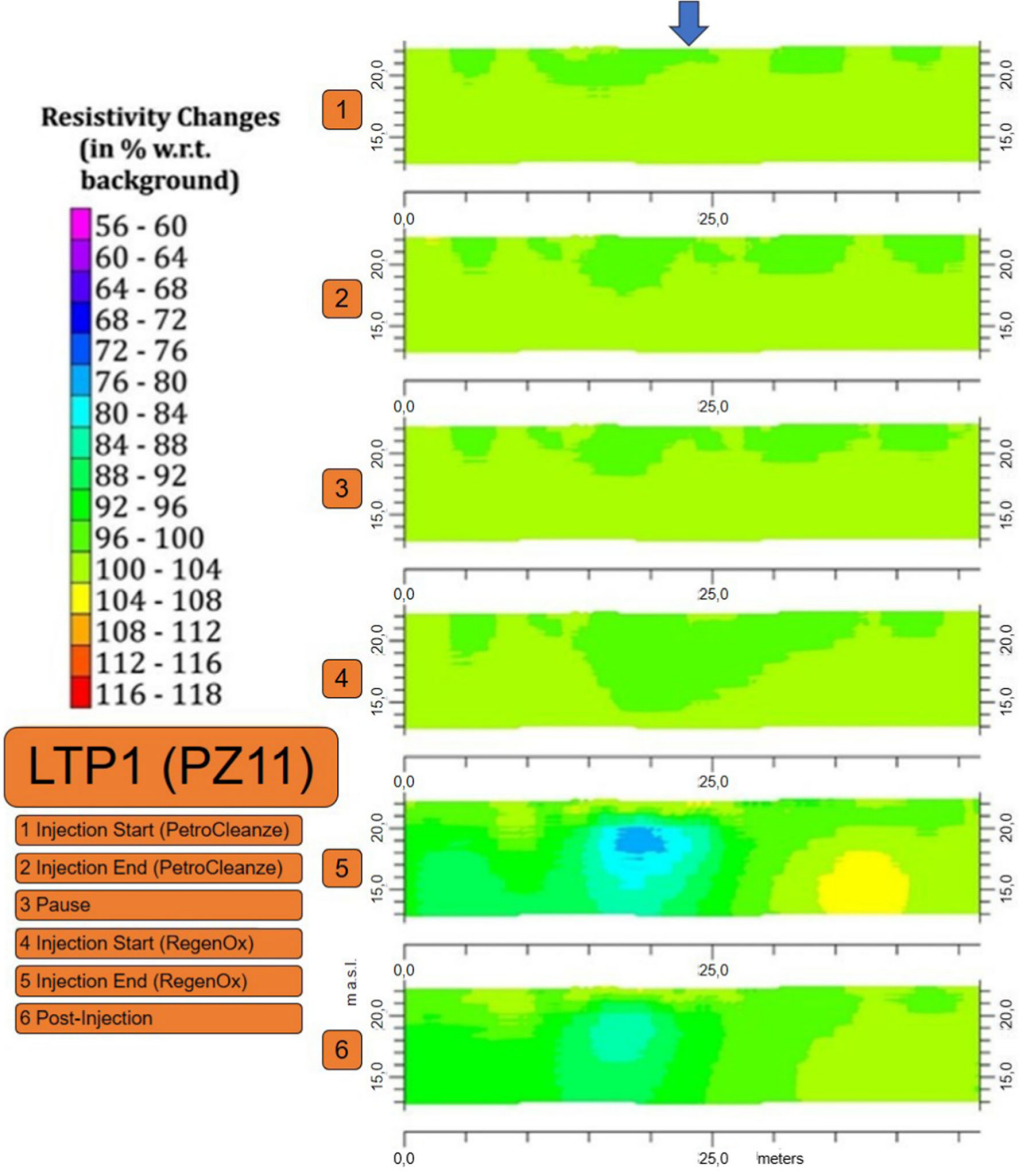
ERT time-lapse results during different field test activities representing the LTP1 line, which covers the PZ11 injection point. The injection point corresponds to the vertical blue arrow

The zones selected for conducting the pilot test differ in terms of the presence or absence of the intermediate clay lens, which influences the hydraulic characteristics of the sediments below the airbase. In addition, the chosen areas recorded the highest contaminant concentrations in groundwater during the historical monitoring campaigns and a sporadic presence of supernatant thicknesses. Performing the test in areas affected by important historical contamination and a different geological setting provided valuable information to evaluate the efficiency of the implemented technology, furnishing insightful evidence about amendment and contaminant behavior in the subsurface. The results of the geophysical investigation reveal the reagent diffusion and the decontamination dynamics (Binley et al. 2010, 2015). The results of the ERT time-lapse surveys carried out in the two areas were expressed as resistivity changes with respect to the background. In particular, the results in correspondence to the long-term persistence 1 (LTP1) line (permeable zone), covering the PZ11 injection point illustrate a good diffusion of the second reagent in the aquifer shown by the light blue color in Fig. 4, as the injected solution is more electrically conductive than the resident groundwater (Morita et al. 2020).

**Fig. 5:**
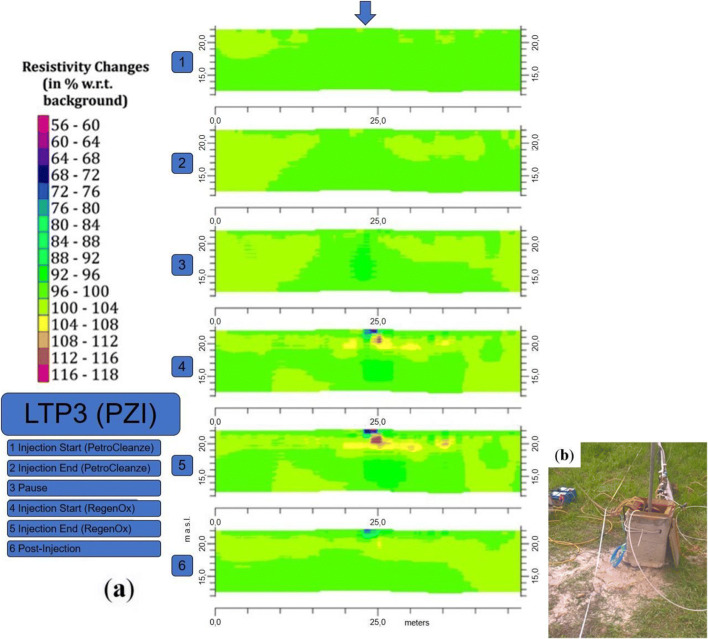
ERT time-lapse results at different stages of reagent application corresponding to the LTP3 line, which covers the PZI injection point. The blue arrow indicates the injection point (**a**), the ascent of the product along the piezometric tube (**b**)

**Fig. 6:**
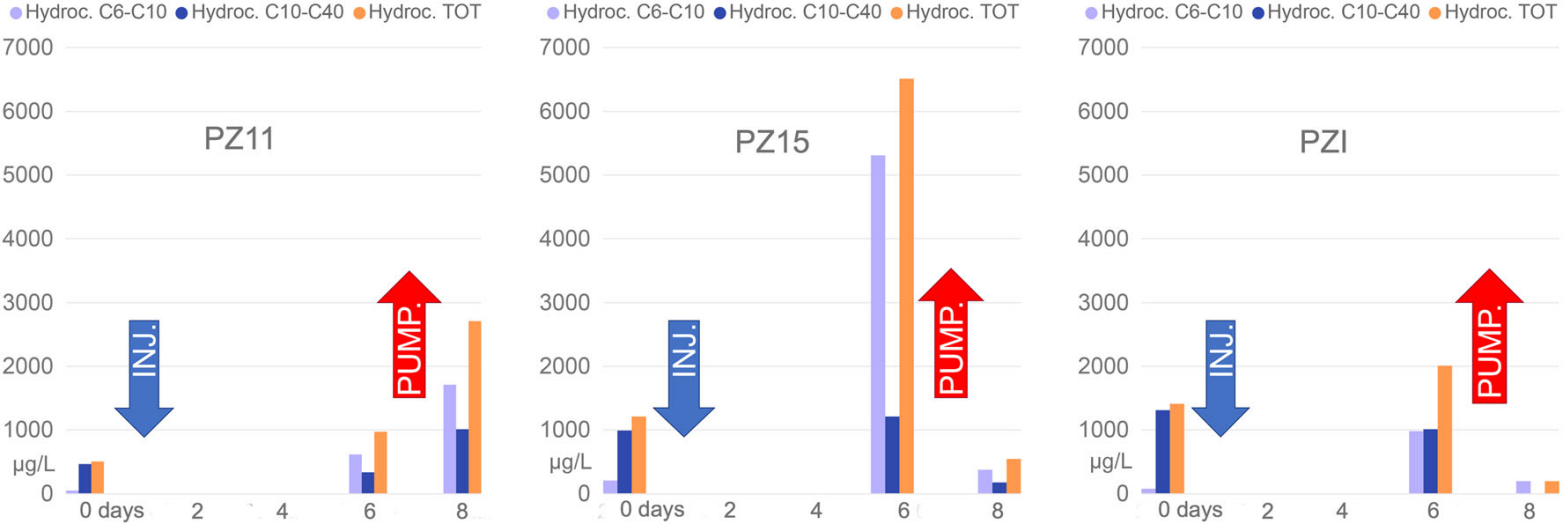
Analysis of water samples recovered during the phases of the pilot test monitoring over time

During the reagent injection activities, the product ascent along the PZI was observed (Fig. 1). The blue shallow anomaly in the ERT time-lapse imaging of the long-term persistence 3 (LTP3) line, covering the area of PZI (having a low permeability), reveals the ascent of the second reagent along the piezometric tube (Fig. 5). On the other hand, the red anomaly indicating the resistivity increase in Fig. 5 is likely to be linked to the mobilization of the contaminants desorbed from the solid matrix (Javanbakht and Goual 2016; Sharma et al. 2020). This assertion is confirmed by the chemical analysis executed on the water samples collected during the implementation of the field test. Monitoring performed on water samples, at the different phases of the pilot test, exhibits a substantial increase in post-application dissolved concentrations, with a subsequent decrease following the pumping activities. The data demonstrate how a considerable mass of contaminants was recovered and how the polluting load was reduced in the area of interest (Fig. 6).

**Fig. 7:**
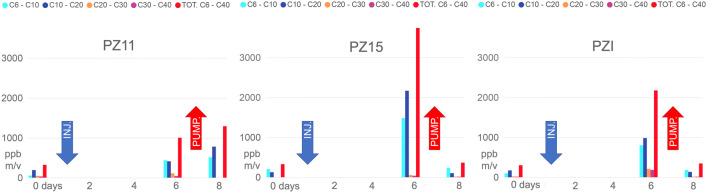
Mass spectrometry and gas chromatography characterization of water samples recovered during the implementation of the experiment at the field scale over time

Speciation analysis, by and large, revealed an increase in the shorter hydrocarbon chains, probably indicating the oxidative effect of the treatment, with partial rupture of longer chains (Tomlinson et al. 2017) (Fig. 7).

## Discussion

The pilot test confirms the mobilization of petroleum products present in the residual phase, which constitutes the secondary source of contamination (Frollini et al. 2016; Teramoto et al. 2020; Verardo et al. 2021). The results obtained at PZ11 infer the occurrence of desorption and oxidation processes even after pumping activities (Besha et al. 2018; Sharma et al. 2020). This evidence provides a valuable indication concerning the site-specific reaction times, and this information will be used to optimize the full-scale intervention configuration. The presence of the hydraulic barrier does not bring issues concerning the possible migration of mobilized contaminants. The extracted water (in which the mobilization of the residual phase was provoked) was conveyed to the existing groundwater treatment plant (Brusseau 2019; Teramoto et al. 2020; Trulli et al. 2016). The monitoring data obtained during and after the test activities show the recovery of a significant mass of contaminants and highlight a reduction of the pollutant load in the application’s area. Data suggest a decrease in the effectiveness of pump-and-treat over time without coupling with technologies that favor the desorption of aged contaminants (Ossai et al. 2020).

The evolution of the concentration of total petroleum hydrocarbons (Fig. 3) detected in the piezometric network confirms that, at present, there is no dissolved plume of contamination. In a portion of the site corresponding to the primary spill events, a residual and insoluble fraction of hydrocarbons with a higher molecular weight remains (Lari et al. 2019b; Ossai et al. 2020; Teramoto et al. 2020; Verardo et al. 2021). This is occasionally “mobilized” and then captured during dynamic sampling activities (Javanbakht and Goual 2016; Sharma et al. 2020).

The results of the pilot test showed how it is possible to mobilize a fraction of this residual hydrocarbon phase (Besha et al. 2018; Sharma et al. 2020). The creation of “reactive” zones in the vadose and in the saturated zones favors the combined action of desorption of residual phase contaminants and oxidation of longer hydrophobic chains (Birnstingl et al. 2014; Lari et al. 2019b; Ossai et al. 2020). The mobilization capacity depends on the area of ​​intervention and is strongly influenced by the different stratigraphic characteristics (Lari et al. 2020; Suthersan et al. 2016).

Geophysical methods represent valuable tools for monitoring the dynamics of decontamination processes that occur within the shallow subsurface (Binley et al. 2010, 2015). ERT measurements, especially performed in time-lapse modality, have furnished intriguing insights into the reactantsʼ repartition in the saturated and unsaturated subsurface, which is greatly influenced by geologic inhomogeneity (Ciampi et al. 2019b). The physical variable of interest, i.e., electrical resistivity, is strongly related to state variables of key environmental interest (Lesmes and Friedman 2005). In the case considered here, the injected solutes (PetroClenze™ and Regenox™) are characterized by a good electrical conductivity (Birnstingl et al. 2014), generally higher than that of resident groundwater. Thus, it is relatively easy to track the injected amendments by using time-lapse ERT. Furthermore, geophysical investigations provide an estimation of the injection radius for each reagent, representing a tool for performance monitoring (Fan et al. 2017). The integrated use of geophysical measurements and chemical analyses is arguably the most effective means of explaining the contaminant-geophysical behavior. This physicochemical model links geophysical signals to contaminant characteristics within contaminated porous media. The coordination of the pilot test through the multidisciplinary and multitemporal data management model and the experimentation at the field scale are relevant indications for optimizing the selected strategy on a full scale.

The biggest constraint of this technique is related to the permeability of the sediments and the presence of preferential flow pathways, which affect the spreading of the product in the subsoil. Fine-grained deposits require low injection pressures to homogeneously redistribute the product or to avoid the rise of amendments along the piezometer. In the future, it is possible to envision combining this remediation technique with a more complex amendment distribution system. Groundwater circulation wells (GCW–IEG) (Ciampi et al. 2019a) could conceivably provide homogeneous product distribution due to the recirculation of fluids in the aquifer, by targeting underground portions classically unaffected by traditional extraction techniques.

## Conclusion

Simultaneous data integration and the multi-source model enabled accurate pilot site selection and the implementation of innovative remediation approaches. Geophysical surveys and groundwater sampling during pilot testing evaluated the effectiveness of the intervention, both in terms of product diffusion capacity and effective reduction of pollutant concentrations. Time-lapse geophysical imagery throughout field experimentation yielded crucial insights, especially about where and how in situ remediation activities impacted distinct parts of the subsoil as a consequence of underground hydraulic inhomogeneity. Chemical analysis showed a significant recovery and a reduction of hydrocarbons, increasing the pumping system efficiency. The physicochemical model, which links geophysical signals to contaminant characteristics within contaminated porous media, was explained through the observation of contaminant-geophysical behavior. The mobilization of the immobile and residual material which constitutes the residual phase of hydrocarbons reveals the limitations of hydraulic barriers. Analysis of all data clearly shows that traditional extraction techniques are ineffective in removing secondary sources of fuel contamination. Our findings suggest that the desorption process using PetroClenze™ and Regenox™ can contribute significantly to the enhancement of hydrophobic pollutantsʼ vacancy. When applied, this two-part reagent generates detergent-like properties, significantly increasing the desorption rates of hydrocarbons bound in saturated soils. Once the hydrocarbons are liberated into the dissolved phase, they are more readily available for removal using physical recovery techniques. The injections of PetroCleanze™ followed by contaminant extraction revealed a significant removal of residual LNAPL, acting as a chemical enhancer for pump and treat.

## Data Availability

The data that support the findings of this study are available from the Italian Air Force and NATO, but restrictions apply to the availability of these data, which were used under license for the current study, and so are not publicly available. Data are however available from the authors upon reasonable request and with permission of the Italian Air Force and NATO.
